# Effects of Dietary Fiber Supplementation on Modulating Uremic Toxins and Inflammation in Chronic Kidney Disease Patients: A Systematic Review and Meta-Analysis of Randomized Controlled Trials

**DOI:** 10.3390/toxins17020057

**Published:** 2025-01-26

**Authors:** Wannasit Wathanavasin, Wisit Cheungpasitporn, Charat Thongprayoon, Tibor Fülöp

**Affiliations:** 1 Division of Nephrology and Hypertension, Department of Medicine, Mayo Clinic, Rochester, MN 55905, USA; wannasit.medical@mfu.ac.th (W.W.); cheungpasitporn.wisit@mayo.edu (W.C.); thongprayoon.charat@mayo.edu (C.T.); 2 Nephrology Unit, Department of Medicine, Charoenkrung Pracharak Hospital, Bangkok Metropolitan Administration, Bangkok 10120, Thailand; 3 Medicine Service, Ralph H. Johnson VA Medical Center, Charleston, SC 29401, USA; 4 Division of Nephrology, Department of Medicine, Medical University of South Carolina, Charleston, SC 29425, USA

**Keywords:** dietary fiber, uremic toxins, chronic kidney disease, inflammation, gut dysbiosis

## Abstract

Emerging evidence supports the beneficial effects of dietary fiber supplementation in alleviating gut dysbiosis, which leads to a reduction in uremic toxins and inflammatory markers in chronic kidney disease (CKD) patients. However, current evidence-based renal nutrition guidelines do not provide recommendations regarding dietary fiber intake. We performed a systematic review and meta-analysis to investigate and highlight the effects of dietary fiber supplementation on modulating uremic toxins and inflammatory markers in individuals with CKD, with or without dialysis. The eligible randomized controlled trials (RCTs) were identified from PubMed, Scopus, and Cochrane Central Register of Controlled trials until 27 November 2024. The results were synthesized using a random-effects model and presented as standardized mean differences (SMDs) with a 95% confidence interval (CI). A total of 21 studies with 700 patients were included. When compared with the control group, dietary fiber supplementation ranging from 6 to 50 g/day, for typically more than 4 weeks, could significantly reduce the levels of serum uremic toxins, including p-cresyl sulfate, indoxyl sulfate, and blood urea nitrogen (SMD −0.22, −0.34, −0.25, respectively, with *p*-values < 0.05), as well as biomarkers of inflammation, including interleukin-6 and tumor necrosis factor alpha (SMD −0.44, −0.34, respectively, with *p*-values < 0.05). These beneficial effects were consistent across different types of fibers and CKD status (with or without dialysis). However, no significant reduction in serum trimethylamine N-oxide, uric acid, and high-sensitivity C-reactive protein levels was observed with dietary fiber intervention. This study would pave the way for prioritizing dietary quality, particularly a fiber-rich diet, beyond the traditional focus on the quantities of protein, energy, and electrolyte restrictions among individuals with CKD.

## 1. Introduction

Dietary interventions have long been a cornerstone in the management of patients with chronic kidney disease (CKD), due to their role in slowing disease progression and preventing its complications. Traditionally, the primary approach has been focused on regulating the intake of protein, sodium, potassium, and phosphorus, along with maintaining an adequate calorie intake [1]. However, emerging evidence suggests that prioritizing dietary quality and diversity, particularly a diet rich in fiber (e.g., plant-based food), is crucial in helping modulate uremic toxin production (through the gut–kidney axis) and promoting overall health in these patients [2,3,4].

**Figure 1:**
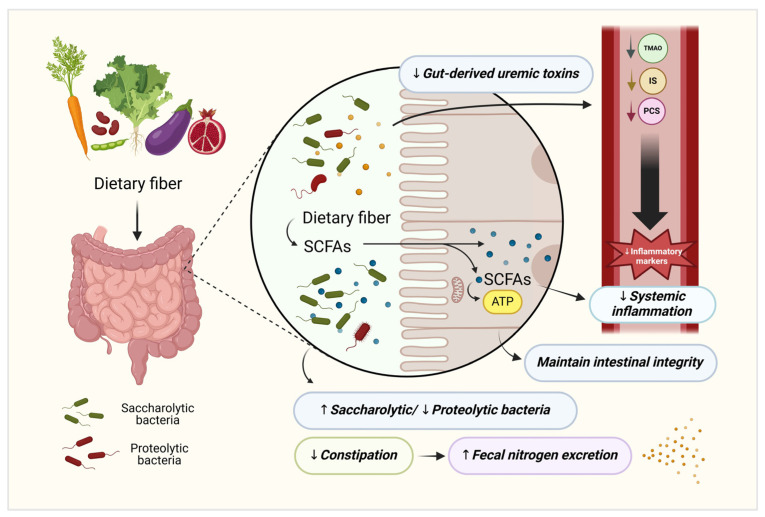
Potential beneficial effects of dietary fiber in patients with CKD. These multifactorial effects include (1) promoting stool output (reducing constipation) and, consequently, (2) increasing nitrogen excretion via fecal mass, (3) reducing the production of gut-derived uremic toxins, oxidative stress, and inflammation from gut microbial modulation (shifting colonic microbial activity from proteolytic to saccharolytic fermentation, and (4) promoting the production of SCFAs, which improves the integrity of the intestinal barrier. ATP, adenosine triphosphate; IS, indoxyl sulfate; PCS, p-cresyl sulfate; SCFAs, short-chain fatty acids; TMAO, trimethylamine N-oxide. (Picture created with biorender.com).

According to the European Food Safety Authority (EFSA), dietary fibers are carbohydrate polymers that are not digestible by the digestive tract enzymes that pass intact to the large intestine, with scientific evidence of their benefits for health [5]. The potential beneficial effects of dietary fiber in patients with CKD, including both non-dialysis dependent (NDD) and dialysis-dependent (DD) patients, have been explained by several mechanisms. These include promoting stool output (laxative effect), improving the integrity of the intestinal barrier, and reducing the production of uremic toxins, oxidative stress, and inflammation from gut microbial modulation (as shown in Figure 1). The increase in bowel transit velocity and the reduction in constipation may reduce blood urea nitrogen (BUN) by altering the urea enterohepatic cycling, increasing nitrogen excretion via fecal mass [6]. In addition, the shift in colonic microbial activity from proteolytic (amino acid-fermenting) to saccharolytic (complex carbohydrate-fermenting) fermentation is considered indicative of a “healthy gut” [7]. In this pattern, saccharolytic predominates, producing short-chain fatty acids (SCFAs), which have positive systemic effects, particularly in protecting against cardiovascular disease (CVD), a major burden in CKD. Conversely, an imbalance where proteolytic fermentation predominates could lead to an increase in the production of gut-derived uremic toxins, including p-cresyl sulfate (PCS), indoxyl sulfate (IS), and trimethylamine N-oxide (TMAO). The accumulation of these uremic toxins is subsequently associated with CKD progression, increased CVD, and mortality in these populations [8,9,10].

It is widely recognized that individuals with CKD often suffer from chronic inflammation and have severely compromised anti-oxidative systems [11], driven by factors not only from gut dysbiosis as mentioned above, but also metabolic disturbance, such as metabolic acidosis hyperuricemia. Dietary fiber also has evident effects in playing a key role in alleviating these conditions. Previous studies [12,13] have shown that high dietary fiber intake or supplementation improves oxidative status and reduces inflammation in individuals with CKD. Additionally, both the US National Health Survey and the China Adult Chronic Disease and Nutrition Surveillance have observed an inverse correlation between serum uric acid levels and dietary fiber intake [14,15]. Therefore, dietary fiber supplementation may be an appealing therapeutic strategy to reduce both uremic toxins and inflammatory markers.

In the general population, the American Dietetic Association [16] recommend a daily fiber intake of 14 g for every 1000 calories per day, or about 25 g for women and 38 g for men each day, to promote health, particularly for protection against cardiovascular disease. Despite the recognized potential benefits of dietary fiber, its consumption among individuals with CKD remains quite low at 5.1 g/day, according to the study by Kwon et al. [17], and current evidence-based nutrition guidelines for CKD, such as the Kidney Disease Outcomes Quality Initiative (KDOQI) guidelines [18], do not offer a strong recommendation regarding dietary fiber intake, including its type, amount, and duration. The aim of this systematic review and meta-analysis is to assess and highlight the effects of dietary fiber supplementation on modulating uremic toxins and inflammatory markers in individuals with CKD, compared to a control group. We seek to provide an updated meta-analysis by combining all available randomized controlled trials (RCTs) to strengthen the evidence, and further explore the effects of fiber supplementation, based on its type, dosage, duration, and solubility, across different stages of CKD.

## 2. Results

### 2.1. Search Results

**Figure 2:**
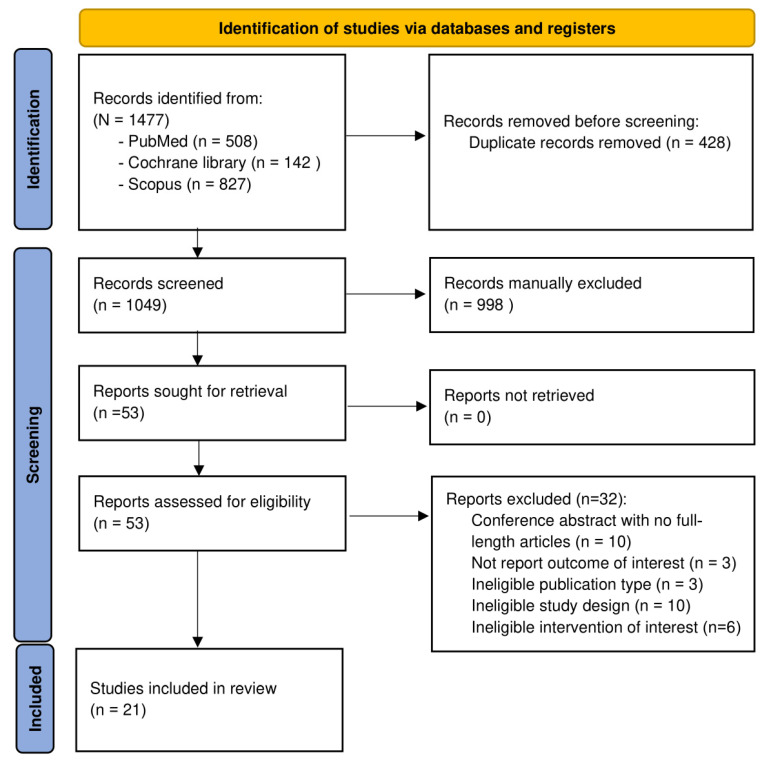
PRISMA 2020 flow diagram.

A total of 1477 potentially relevant articles were initially identified through the database search (from Pubmed [n = 508], Cochrane library [n = 142], and Scopus [n = 827]). After removing 428 duplicate articles, 1049 article titles and abstracts were screened based on the inclusion and exclusion criteria. The remaining 53 articles were selected for full-text review, of which 32 were excluded (the reasons for full-text exclusion are detailed in Figure 2). Finally, a total of 21 articles were included in the systematic review and meta-analysis (Figure 2).

### 2.2. Study Characteristics

Table 1 provides detailed characteristics and key features of the individual studies. Among the 21 RCTs included, 7 studies recruited NDD-CKD patients [19,20,21,22,23,24,25], while 14 involved DD-CKD patients (9 studies on hemodialysis (HD) [26,27,28,29,30,31,32,33,34] and 5 studies on peritoneal dialysis (PD) [35,36,37,38,39]). A total of 10 studies were cross-over designs and 11 were parallel. A total of 700 individuals with CKD were included, comprising 299 NDD-CKD (42.71%) and 401 DD-CKD (57.29%) patients. Seven studies were conducted in Asia (China, Iran, and Thailand), five in North America (Canada and the USA), five in South America (all in Brazil), two in Europe (both in Belgium), and two in Africa (South Africa and Sudan). These studies yielded a total sample of five continents (Asia: 29%, South America: 26.71%, North America: 23.29%, Africa: 13.57%, and Europe: 7.43%). Of the included patients, the mean age was 51.9 years, and 55.78% were male. The mean dialysis vintage was 46.1 months among DD-CKD patients. The type of fiber supplementation included non-starch polysaccharide (NSP), resistant oligosaccharide (RO), resistant starch (RS), and mixed forms, with RO and RS being the most frequently used. The daily dosages ranged from 6 to 50 g, and the supplementation duration ranged 4 to 16 weeks.

### 2.3. Risk of Bias Assessment

**Figure 3:**
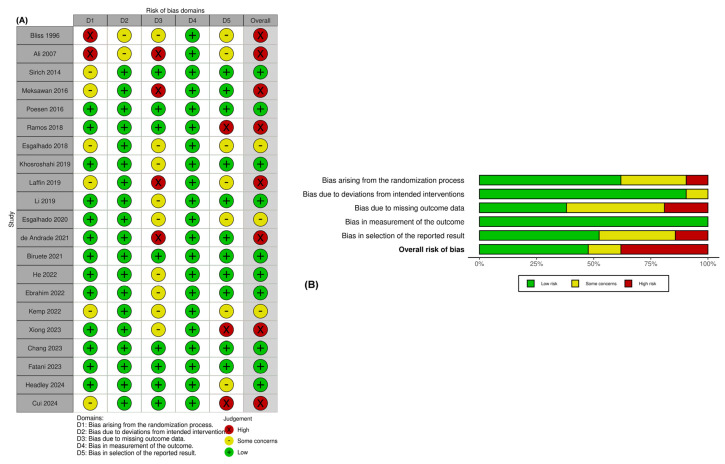
Risk of bias assessment of included RCTs; (**A**) traffic light plot, (**B**) weighted summary plot of the overall type of bias [19,20,21,22,23,24,25,26,27,28,29,30,31,32,33,34].

According to the Cochrane risk of bias assessment tool 2 (RoB2) [40], ten studies were identified as having a low risk of bias, eight had a high risk of bias, and three had some concerns of bias. Figure 3 provides details on the rating of each RoB2 domain and presents the RoB2 evaluations of included studies. The overall assessment indicated a moderate bias (Figure 3A,B).

### 2.4. Effects of Dietary Fiber Supplementation on Uremic Toxins Outcomes (Table 2)

#### 2.4.1. Serum p-Cresyl Sulfate (PCS)

**Figure 4:**
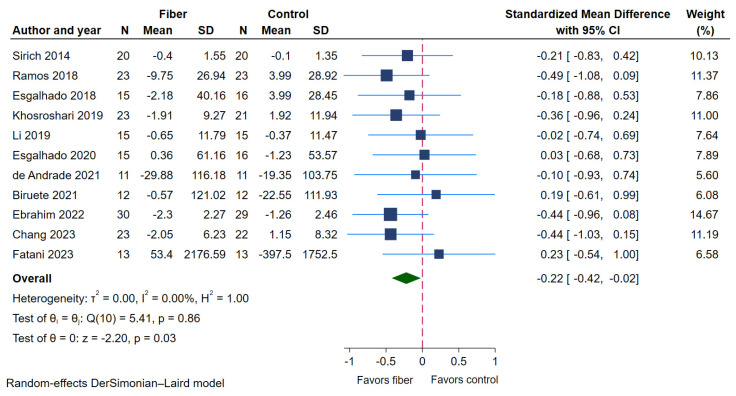
Forest plot of SMD (95% CI) showing the effect of dietary fiber supplement on serum PCS [21,22,23,27,28,29,31,32,34,36,37].

Eleven RCTs [21,22,23,27,28,29,31,32,34,36,37] (200 patients in the dietary fiber supplementation group and 198 patients in the control group) measured serum PCS as outcome. Compared with the control group, dietary fiber supplementation significantly reduced serum PCS (standardized mean differences (SMD) −0.22, 95% confidence interval (CI) −0.42 to −0.02; *p* = 0.03; Figure 4, Table 2). There was no obvious heterogeneity (I^2^ 0%, *p* = 0.86; Figure 4, Table 2). The results of the subgroup analyses are presented in Table 3. In summary, the findings demonstrated that the reduction in serum PCS levels was more significant in the parallel study design (SMD −0.37, 95% CI −0.61 to −0.31, *p* for interaction = 0.04) compared to the crossover design. There were no significant effects observed in any of the other subgroups, including race, CKD status, study risk of bias, type, dosage, solubility, and duration of fiber supplementation (*p* for interaction = 0.60, 0.08, 0.72, 0.55, 0.75, 0.36, 0.08, respectively). However, in terms of CKD status, the results showed a trend indicating that NDD-CKD patients seemed to have a more obvious lowering effect on serum PCS than DD-CKD patients (Table 3).

**Table 2:** Summary of results for primary and secondary outcomes.

Outcomes	No. of Studies	No. of Patients			SMD (95% CI)	I^2^	GRADE Evidence
		Total	Intervention	Control			
**Primary Outcomes**: **Uremic toxins**							
Serum PCS	11	398	200	198	−0.22 (−0.42 to −0.02)	0%	⨁⨁⨁⨁ High
Serum IS	11	398	200	198	−0.34 (−0.57 to −0.12)	19.92%	⨁⨁⨁⨁ High
BUN	11	398	200	198	−0.25 (−0.48 to −0.03)	21.39%	⨁⨁◯◯ Low
Serum TMAO	4	135	67	68	+0.05 (−0.29 to 0.39)	0%	⨁◯◯◯ Very low
Serum uric acid	4	118	60	58	−0.18 (−0.61 to 0.25)	25.37%	⨁◯◯◯ Very low
**Secondary Outcomes**: **Inflammatory markers**							
Serum IL-6	7	265	131	134	−0.44 (−0.73 to −0.16)	24.47%	⨁⨁⨁◯ Moderate
Serum hs-CRP	5	218	110	108	−0.01 (−0.38 to 0.36)	47.76%	⨁◯◯◯ Very low
Serum TNF-α	4	157	78	79	−0.34 (−0.66 to −0.02)	0%	⨁◯◯◯ Very low

Abbreviation: BUN, blood urea nitrogen; CI, confident interval; hs-CRP, high sensitivity C-reactive protein; IL-6, interleukin-6; IS, indoxyl sulfate; PCS, p-cresyl sulfate; SCFAs; TMAO, trimethylamine N-oxide; TNF-α, tumor necrotic factor alpha.

**Table 3:** Subgroup analyses examining the effect of dietary fiber supplementation on serum PCS.

Subgroup Analyses	No. of Studies	SMD (95% CI)	*p*-Values	Assessment of Heterogeneity		*p* for Interaction
				I^2^ Index	*p*-Value	
**CKD status**						0.08
NDD-CKD	3	−0.45 (−0.78 to −0.13)	0.01	0%	0.99	
DD-CKD	8	−0.08 (−0.33 to 0.17)	0.51	0%	0.95	
**Study design**						0.04
Crossover	5	0.06 (−0.28 to 0.40)	0.71	0%	0.98	
Parallel	6	−0.37 (−0.61 to −0.13)	<0.01	0%	0.97	
**Fiber dosage**						0.75
≤15 g/day	7	−0.24 (−0.49 to 0.00)	0.05	0%	0.6	
>15 g/day	4	−0.18 (−0.52 to 0.17)	0.32	0%	0.87	
**Race**						0.60
Asian	3	−0.30 (−0.67 to 0.06)	0.10	0%	0.67	
Non-Asian	8	−0.19 (−0.42 to 0.05)	0.12	0%	0.74	
**Study risk of bias**						0.72
Low	7	−0.22 (−0.46 to 0.02)	0.07	0%	0.68	
Some concerns	2	−0.08 (−0.57 to 0.42)	0.77	0%	0.69	
High	2	−0.36 (−0.84 to 0.12)	0.14	0%	0.45	
**Intervention duration**						0.08
<8 week	6	−0.03 (−0.32 to 0.27)	0.86	0%	0.94	
≥8 week	5	−0.38 (−0.64 to −0.11)	0.03	0%	0.88	
**Type of fiber**						0.55
NSP	1	−0.44 (−0.96 to 0.08)	0.10	-	-	
RO	4	−0.26 (−0.59 to 0.07)	0.12	0%	0.46	
RS	5	−0.18 (−0.49 to 0.12)	0.24	0%	0.95	
Mixed	1	0.23 (−0.54 to 1.00)	0.56	-	-	
**Fiber solubility**						0.36
Non-water soluble	6	−0.13 (−0.41 to 0.15)	0.37	0%	0.89	
Water soluble	5	−0.31 (−0.59 to −0.04)	0.03	0%	0.86	

Abbreviation: NSP, non-starch polysaccharide; RO, resistant oligosaccharide; RS, resistant starch; SMD, standardized mean difference.

#### 2.4.2. Serum Indoxyl Sulfate (IS)

**Figure 5:**
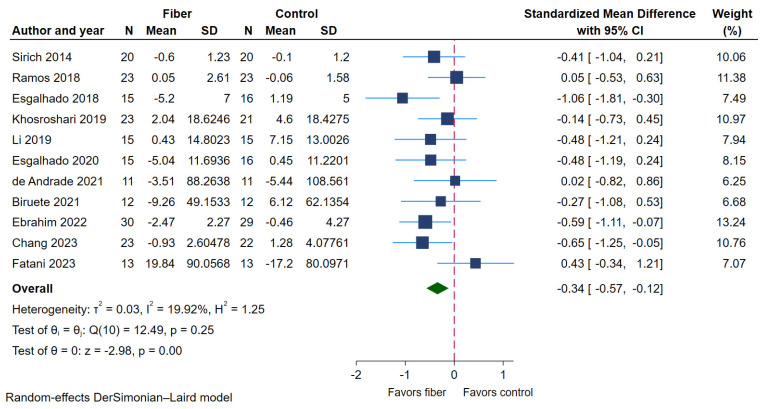
Forest plot of SMD (95% CI) showing the effect of dietary fiber supplement on serum IS [21,22,23,27,28,29,31,32,34,36,37].

Out of 21 RCTs, 11 RCTs [21,22,23,27,28,29,31,32,34,36,37] (200 patients in the dietary fiber supplementation group and 198 patients in the control group) reported data on serum IS. Compared with the control group, dietary fiber supplementation significantly reduced serum IS (SMD −0.34, 95% CI −0.57 to −0.12; *p* < 0.01; Figure 5, Table 2). The heterogeneity between studies was mild (I^2^ 19.92%, *p* = 0.25; Figure 5, Table 2). The results from the subgroup analyses (Table 4) revealed that study design, race, CKD status, study risk of bias, type, dosage, solubility, and duration of fiber supplementation did not significantly influence the pooled effect estimate (*p* for interaction = 0.27, 0.66, 0.10, 0.73, 0.18, 0.72, 0.62, 0.84, respectively).

#### 2.4.3. Serum Trimethylamine N-Oxide (TMAO)

Four RCTs [25,33,34,39] (67 patients in the dietary fiber supplementation group and 68 patients in the control group) reported data on serum TMAO. The results showed that dietary fiber supplementation did not significantly reduce serum TMAO (SMD 0.05, 95% CI −0.29 to 0.39; *p* = 0.78; Table 2) compared with the control group. There was no obvious heterogeneity (I^2^ 0%, *p* = 0.56; Table 2).

#### 2.4.4. Blood Urea Nitrogen (BUN)

Eleven RCTs [19,21,23,25,26,27,28,29,30,35,37] (200 patients in the dietary fiber supplementation group and 198 patients in the control group) reported data on BUN. The results demonstrated that dietary fiber supplementation significantly reduces BUN (SMD −0.25, 95% CI −0.48 to −0.03; *p* = 0.03; Table 2) compared with the control group. The heterogeneity between studies was mild (I^2^ 21.39%, *p* = 0.24; Table 2). Subgroup analyses revealed that the beneficial effects of dietary fiber on reducing BUN was consistent across all subgroups, including study design, race, CKD status, study risk of bias, type, dosage, solubility, and duration of fiber supplementation (*p* for interaction = 0.65, 0.86, 0.79, 0.12, 0.25, 0.15, and 0.52, respectively; Table S1). However, regarding CKD status, the results indicated a trend suggesting that DD-CKD patients had a more pronounced reduction in BUN levels than NDD-CKD patients (Table S1).

#### 2.4.5. Serum Uric Acid

Serum uric was reported in 4 RCTs [26,29,30,38] (60 patients in the dietary fiber supplementation group and 58 patients in the control group). The results showed that dietary fiber supplementation did not significantly reduce serum uric acid (SMD −0.18, 95% CI −0.61 to 0.25; *p* = 0.40; Table 2) compared with the control group. The heterogeneity between studies was moderate (I^2^ 25.37%, *p* = 0.26; Table 2).

### 2.5. Effects of Dietary Fiber Supplementation on Inflammatory Markers (Table 2)

#### 2.5.1. Serum Interleukin-6 (IL-6)

Serum IL-6 was reported in 7 studies [21,23,25,28,30,31,37] (131 patients in the dietary fiber supplementation group and 134 patients in the control group). The results showed that dietary fiber supplementation significantly reduces serum IL-6 (SMD −0.44, 95% CI −0.73 to −0.16; *p* < 0.01; Table 2) compared with the control group. The heterogeneity between studies was mild (I^2^ 24.47%, *p* = 0.24 Table 2). Subgroup analyses revealed that the beneficial effect of dietary fiber on reducing serum IL-6 was consistent across all subgroups, including study design, race, CKD status, study risk of bias, type, dosage, solubility, and duration of fiber supplementation (*p* for interaction = 0.96, 0.78, 0.42, 0.84, 0.75, 0.84, 0.75, 0.34, respectively; Table S2).

#### 2.5.2. Serum Tumor Necrotic Factor Alpha (TNF-α)

Serum TNF-α was reported in 4 studies [23,25,30,37] (78 patients in the dietary fiber supplementation group and 79 patients in the control group). The results showed that dietary fiber supplementation significantly reduces serum TNF-α (SMD −0.34, 95% CI −0.66 to −0.02; *p* = 0.03; Table 2) compared with the control group. There was no obvious heterogeneity (I^2^ 0%, *p* = 0.66; Table 2).

#### 2.5.3. Serum High-Sensitivity C-Reactive Protein (hs-CRP)

Serum hs-CRP was reported in 5 studies [21,23,28,29,37] (110 patients in the dietary fiber supplementation group and 108 patients in the control group). The results showed that dietary fiber supplementation did not reduce serum hs-CRP (SMD −0.01, 95% CI −0.38 to 0.36; *p* = 0.96; Table 3) compared with the control group. The heterogeneity between studies was moderate (I^2^ 47.76%, *p* = 0.10; Table 2).

### 2.6. Sensitivity Analysis

In order to ensure the reliability of the present meta-analysis, we performed a sensitivity analysis to assess the robustness of the pooled SMDs by sequentially excluding each study (leave-one-out method). The results indicated that the heterogeneity among the studies did not change significantly with respect to the outcomes of both uremic toxins and inflammatory markers.

### 2.7. Meta-Regression

Meta-regression analyses of outcomes involving more than 10 studies (i.e., serum PCS, serum IS, and BUN) showed that the effects of dietary fiber supplementation on a reduction in serum PCS, IS, and BUN levels in individuals with CKD was not associated with the year of publication, mean participant age, study sample size, dialysis vintage in DD-CKD, daily dose, or the duration of supplementation (Table S3). However, one significant exception was observed: the reduction in serum PCS showed a significant correlation with a larger study sample size (*β* = −0.015; *p* = 0.029; R^2^ = 0%).

### 2.8. Assessment of Publication Bias and Strength of Evidence

Publication bias was assessed using visual inspection of funnel plots and Egger’s regression model. The funnel plots for serum PCS (Figure S1) and serum IS (Figure S2) outcomes in the included studies were symmetrical. The Egger’s test did not indicate significant publication bias for both outcomes (*p* = 0.09 for serum PCS and 0.58 for serum IS). However, for the BUN outcome, the funnel plot was asymmetrical (Figure S3), and the Egger’s test showed significant publication bias (*p* = 0.006). The strength of evidence for SMD of serum PCS and IS levels compared between dietary fiber supplementation and control were graded as high, for BUN as low, and for serum TMAO, uric acid as very low (Table 2). Further details about the grading evaluation of both primary and secondary outcomes are provided in Table S4.

## 3. Discussion

This systematic review and meta-analysis examined and updated the effects of dietary fiber supplementation on modulating uremic toxins and inflammatory markers in individuals with CKD by summarizing the results from twenty-one RCTs. Our findings indicated that dietary fiber supplementation could significantly reduce the levels of serum uremic toxins, including PCS, IS, and BUN, as well as biomarkers of inflammation, including IL-6 and TNF-α, when compared with the control group. These beneficial effects were consistent across all subgroups, including race, CKD status, study risk of bias, type, dosage, solubility, and duration of fiber supplementation. However, no significant reduction in serum TMAO, uric acid, and hs-CRP levels was observed with dietary fiber intervention.

The gut–kidney axis refers to the interaction between the gastrointestinal tract and the kidneys, and its disorders have become increasingly recognized in individuals with CKD due to their association with the production of gut-derived uremic toxins (GDUTs) and the progression of the disease [41]. In addition to the disease itself, the type of dietary intake plays a crucial role in the production of these toxins [42]. In CKD patients, proteolytic bacteria dominate the colon and metabolize dietary proteins, generating various byproducts, including PCS and IS, the two prototypes of GDUTs [43]. As such, fermentation of phenylalanine and tyrosine subsequently results in the synthesis of p-cresol, which is finally converted to PCS, while tryptophan undergo fermentation leads to produce indole, which is then metabolized to IS in the liver [44]. These metabolites are extensively researched for their reduction due to their persistent elevation being linked to several adverse effects, such as increased cardiovascular disorders and all-cause mortality in both NDD-CKD and DD-CKD [10,45,46]. As the nature of these toxins is protein-bound with high-binding capacity, it is difficult to remove them from systemic circulation via conventional dialysis [47]. Recently, dietary interventions aimed at restoring gut eubiosis and mitigating these toxins have been widely proposed. Apart from low protein intake, our findings indicate that dietary fiber intervention has a promising favorable effect on modulating these toxins, primarily through gut microbial modulation, as illustrated in Figure 1. Consistent with parts of the previous meta-analysis by Wu et al. [48], this study revealed that dietary fiber intake effectively reduces serum PCS levels (weighted mean difference (WMD) −16.16, 95%CI: −23.82 to −8.50), but does not significantly affect serum IS levels (WMD −0.21, 95%CI: −2.35 to 1.93). Nevertheless, this meta-analysis has several limitations, such as a limited number of studies, focus only on pre-dialysis CKD, a short follow-up period, and the lack of subgroup analysis. Another meta-analysis by Yang et al. [3] further validated the results, expanding the CKD population to include individuals receiving dialysis and focusing exclusively on RCT designs. It demonstrated that dietary fiber intervention can significantly lower PCS and IS levels in CKD patients. However, limitations still persisted due to moderate heterogeneity in the findings and a primary focus on non-viscous and soluble types of fibers. Eventually, our analysis provided more conclusive results on the effects of dietary fiber supplementation in reducing serum PCS and IS levels, with less heterogeneity (I^2^ = 0%) and a variety of fiber types (i.e., NSP, RO, RS, and mixed), offering high certainty of the Grading of Recommended Assessment, Development, and Evaluation approach (GRADE) evidence (Table 2).

There are several important aspects of the subgroup analysis of serum PCS and IS outcomes that should be highlighted. Although crossover designs are frequently used in nutrition research because they require smaller sample sizes than parallel designs to achieve similar statistical power, they may introduce carryover effects and potential bias [49]. As a result, we performed a pre-specified subgroup analysis and found that study design appears to be a significant factor. The analysis revealed a more pronounced reduction effect in parallel trials than in crossover trials, particularly for serum PCS (*p* for interaction = 0.04; Table 3). It is important to note that the treatment effect in crossover trials may not solely be attributed to the intervention itself, but also to interactions between the treatment and study periods, as most of the included crossover studies report combined data from both the treatment and control periods. Compared to DD-CKD, the reduction in serum PCS and IS appears to be more pronounced in NDD-CKD (Table 3 and Table 4). The explanation behind this result may lie in the contrasting recommendations of daily protein intake between these two populations [18]. NDD-CKD patients are advised to limit their protein consumption (0.6–0.8 g/kg/day) to slow disease progression, whereas DD-CKD patients need to consume more protein (1.0–1.2 g/kg/day) to compensate the inevitable loss of amino acids and proteins during dialysis treatment. The complexities of NDD-CKD and DD-CKD populations are distinct and may influence the effectiveness of dietary fiber supplementation. For example, NDD-CKD individuals typically have lower baseline levels of uremic toxins and follow a protein-restricted diet, which may enhance the observed effects of fiber on reducing PCS and IS levels. Conversely, DD-CKD individuals experience significant protein losses during dialysis sessions, which could alter their response to dietary fiber supplementation. While subgroup analyses showed trends suggesting differences in response between these populations, the lack of statistically significant interactions (*p* for interaction > 0.05) highlights the need for further research to elucidate these differences. According to the subgroup analysis of fiber solubility types, water-soluble fibers (e.g., inulin and fructo-oligosaccharides (FOS)) appear to be more effective in decreasing GDUT levels than insoluble fibers (e.g., resistant starch). However, it is important to note that the *p* for interaction was >0.05, indicating no statistically significant difference in the observed effect. Despite this, the distinct biological mechanisms associated with different fiber types warrant attention. Our study evaluated the effects of dietary fiber supplementation across a wide range of dosages, from 6 to 50 g/day, as no definitive recommendations for an optimal dosage exist. Subgroup analysis (≤15 g/day vs. >15 g/day) and meta-regression revealed no significant dose-dependent differences, highlighting the general efficacy of dietary fiber within this range. Soluble fibers, such as inulin and FOS, are known to promote saccharolytic fermentation by gut microbiota, resulting in the production of short-chain fatty acids (SCFAs), which can exert anti-inflammatory effects and improve gut barrier function. In contrast, insoluble fibers, including resistant starch, primarily enhance stool bulk and motility, potentially reducing the reabsorption of gut-derived uremic toxins. While our data suggest a general beneficial effect of dietary fiber supplementation regardless of type, these mechanistic differences emphasize the need for further studies to evaluate specific fiber types and their optimal applications in individuals with CKD.

Dietary fiber encompasses a heterogeneous group of carbohydrate polymers with distinct physicochemical properties, including solubility, viscosity, and fermentability, which significantly influence their biological effects [2,3,4]. Soluble fibers, such as inulin and FOS, tend to promote saccharolytic fermentation by gut microbiota, producing beneficial metabolites like SCFAs. In contrast, insoluble fibers, including resistant starch, mainly enhance stool bulk and bowel motility. The diversity in fiber types is critical in modulating the gut–kidney axis, which plays a central role in CKD pathophysiology. For instance, dietary fibers influence gut microbiota composition, shifting microbial activity from proteolytic to saccharolytic fermentation, thereby reducing the production of gut-derived uremic toxins (PCS and IS) associated with CKD progression. This heterogeneity was addressed in our meta-analysis through subgroup analyses based on fiber type and solubility, which revealed that water-soluble fibers had a more pronounced effect in reducing uremic toxins and inflammatory markers compared to non-water-soluble fibers [2,3,4].

TMAO is a gut-derived uremic toxin with a low molecular weight and high-water solubility, elevated in those with impaired kidney function. Unlike PCS and IS, it is efficiently removed by dialytic clearance. The interest in TMAO has increased in the nephrology community because of its association with CVD, which in individuals with CKD is the leading cause of mortality [50,51,52]. Consistent with the previous study by Miao et al. [53], our meta-analysis revealed that dietary fiber supplementation does not lower serum TMAO levels among CKD patients (SMD + 0.05, *p* = 0.78; Table 2). The lack of significance may be attributed to (1) the wide variation in plasma TMAO levels, both inter- and intra-individually, which are also strongly influenced by age and gender [54,55], and (2) inadequate dosage and the relatively short supplementation period. Most of the included studies that addressed this outcome [25,33,34,39] administered a dose of less than 15 g/day, with follow-up time limited to 4 weeks, which may have been insufficient to alter gut microbiota in individuals with CKD.

Besides the GDUTs mentioned earlier, urea, a long-forgotten molecule, has been implicated in causing gut dysbiosis in individuals with CKD. The accumulation of urea in body fluids results in its massive influx into the intestines, where bacterial urease breaks it down into ammonia. Ammonia is subsequently converted to ammonium hydroxide, which raises the pH in the intestinal lumen, damages the intestinal epithelial tight junctions, and impairs its mucosal barrier function [56]. Urea also serve as an alternative substrates for bacterial species that normally utilize indigestible carbohydrates [57]. The results from our meta-analysis found that dietary fiber supplementation significantly reduced BUN (SMD −0.25; *p* = 0.03; Table 2), compared with the control group, and were consistent across all subgroups, including study design, race, CKD status, study risk of bias, type, dosage, solubility, and duration of fiber supplementation. However, there was a trend suggesting that DD-CKD patients had a more pronounced reduction in BUN levels compared to NDD-CKD patients [19,58,59]. This finding aligns with the different dietary recommendations for protein intake in these two populations. NDD-CKD patients are advised to limit protein intake (0.6–0.8 g/kg/day) to slow disease progression, whereas DD-CKD patients require higher protein intake (1.0–1.2 g/kg/day) to compensate for protein losses during dialysis. Dietary fiber may reduce BUN levels in NDD-CKD by promoting nitrogen excretion as microbial proteins in the stool, whereas in DD-CKD, the effect may be enhanced by the higher baseline levels of BUN due to dialysis-associated protein losses. Moreover, alterations in colonic microbial activity enhance the production of SCFAs, which bind to ammonia and prevent its reabsorption by the liver, thereby disrupting the enterohepatic circulation [60]. Nevertheless, caution is warranted in interpreting these results due to potential confounding factors such as variations in dietary compliance, baseline fiber intake, and study designs.

Hyperuricemia (HUA) is common in patients with CKD, affecting over 60% in the advanced stages of disease [61]. Besides renal underexcretion, it is also recognized as a lifestyle-related condition, particularly the excessive dietary intake of purines. Research on animals [62,63] strongly suggests that dietary fiber may play a role in preventing and controlling HUA due to excessive purine consumption, regardless of kidney function. Furthermore, data from the National Health and Nutrition Examination Survey (NHANES) conducted between 2009 and 2014 showed that dietary intakes of total fiber and cereal fiber were inversely associated with the risk of hyperuricemia. The odds ratios for the highest versus lowest quartile intakes were 0.58 (95% CI 0.46 to 0.74) for total fiber and 0.61 (95% CI 0.52 to 0.74) for cereal fiber, based on a U.S. adult population of 12,869 participants [14]. However, there is limited information regarding the relationship between dietary fiber intake and hyperuricemia in the CKD population. Based on our meta-analysis of four RCTs, we found that dietary fiber supplementation did not significantly reduce serum uric acid (SMD −0.18, *p* = 0.40; Table 2). Possible explanations for these non-significant results include a relatively small sample size, which may not have provided adequate statistical power, and the sole focus on the DD-CKD population, which typically has a high protein intake that contains substantial amounts of purines [64], potentially obscuring the fiber’s effect. Future RCTs with larger sample sizes and extended follow-up periods are necessary to explore this issue further.

In patients with CKD, gut microbiota disruption also results in systemic inflammation, with GDUTs such as PCS, IS, and TMAO being key contributors to the production of reactive oxygen species (ROS) and proinflammatory cytokines. This chronic inflammation is often exacerbated by disease progression and the initiation of dialysis, resulting in increased levels of inflammatory markers. Indeed, this inflammatory state is linked to adverse outcomes such as malnutrition, protein-energy wasting, sarcopenia, and several comorbidities, including cardiovascular disease [65,66]. Improving dietary quality, particularly by incorporating fiber-rich, nutritious foods, could offer a promising strategy for addressing inflammation in these individuals. As demonstrated in our study, we assessed the effects of dietary fiber on inflammation and found it significantly lowered inflammatory biomarkers, including IL-6 and TNF-α (SMD −0.44, −0.34, respectively, with *p*-values < 0.05), when compared to the control group. Consistent with NHANES III data [13], high dietary fiber intake is associated with a reduced risk of inflammation, though different biomarkers may be involved. This association appears to be stronger in patients with CKD than in healthy individuals. Several mechanisms have been proposed to explain how high dietary fiber intake leads to lower inflammatory markers. Besides decreasing GDUTs (i.e., PCS, IS, and BUN) in our meta-analysis, dietary fiber has been linked to higher levels of anti-inflammatory adiponectin in circulation [67]. In addition, modulation of gut microbiota, including enrichment of SCFA-producing bacteria, may help alleviate systemic inflammation in CKD patients by regulating immune responses and anti-inflammatory pathways through T cell regulation and inflammatory gene modulation [7,68]. However, the lack of a significant reduction in hs-CRP in our study suggests that IL-6 and TNF-α levels may be more sensitive biomarkers to dietary fiber intake than hs-CRP levels, similar to the data observed in post-menopausal women from Ma et al. [69].

The present meta-analysis has several strengths. It offers a comprehensive and up-to-date review of RCTs that assess the effects of dietary fiber supplementation on a broad range of uremic toxins (including PCS, IS, TMAO, BUN, and uric acid) and inflammatory biomarkers (including IL-6, TNF-α, and hs-CRP) in CKD patients with or without dialysis. With data from 21 RCTs and a total of 700 CKD patients, our study is one of the largest contributions to the field of renal nutrition regarding fiber supplementation’s effects on these outcomes. By focusing exclusively on RCTs, we ensure a high quality of evidence for the causal relationship, particularly regarding PCS and IS outcomes. Furthermore, we conducted subgroup analyses and meta-regression to identify several key moderators of the relationship between dietary fiber and outcomes of interest.

However, it is acknowledged this study has some limitations. Firstly, the baseline dietary fiber intake was not accounted for due to the paucity of reporting in the included studies. Therefore, the fiber dosage in the intervention group may not reflect the true difference in fiber intake between the two groups. Secondly, concerns arise regarding the heterogeneity of the studies and publication bias in certain outcomes, such as BUN and serum hs-CRP. The studies varied in design, participant characteristics, and the type, dose, and duration of interventions, contributing to heterogeneity and limiting comparability on these outcomes. Moreover, differences in compliance and adherence of diets were not taken into account as a source of heterogeneity. Thirdly, crossover studies were incorporated in the meta-analysis as though they were parallel designs, without accounting for the different intervention periods. Nevertheless, we carried out a pre-specified subgroup analysis based on study design to assess whether variations in design affected the pooled effect estimates. Lastly, the clinical benefits of reducing these metabolites were not evaluated in this study. Given the toxic effects of these uremic toxins and inflammatory markers, lowering their levels could positively impact renal progression, cardiovascular disease, and mortality in CKD patients, as previously discussed. Future large-scale, well-conducted RCTs with extended follow-up periods and a robust multifactorial design are needed to assess clinical outcomes.

## 4. Conclusions

In conclusion, the current systematic review and meta-analysis of RCTs highlighted that dietary fiber supplementation is a potential treatment for lowering various uremic toxins and inflammatory markers in both NDD-, and DD-CKD patients. This investigation demonstrated that dietary fiber supplementation ranging from 6 to 50 g/d, for typically more than 4 weeks, holds great potential in lowering GDUTs, including serum PCS and IS, with high certainty of evidence, which could help alleviate adverse clinical outcomes, especially CVD and mortality. In addition, it also had a significant beneficial effect on BUN reduction, albeit with a low quality of evidence. In terms of inflammatory markers, fiber supplementation can significantly reduce both serum IL-6 and TNF- α, with moderate and very low certainty of evidence, respectively. This research would pave the way for prioritizing dietary quality, particularly a fiber-rich diet, beyond the traditional focus on the quantities of protein, energy, and electrolyte restrictions among individuals with CKD.

## 5. Materials and Methods

This systematic literature review was performed in accordance with the 2020 Preferred Reporting Items for Systematic Reviews and Meta-Analyses (PRISMA) guidelines for reporting interventions (Table S5). The protocol was pre-registered in the PROSPERO database (registration number CRD42024623503).

### 5.1. Searching Strategy

Two independent reviewers (W.W. and W.C.) systematically searched all randomized controlled trials (RCTs) evaluating the effects of dietary fiber supplementation on modulating uremic toxins in patients with chronic kidney disease. A database search was carried out on 27 November 2024, using PubMed, Scopus, and Cochrane Central Register of Controlled Trials (CENTRAL). No date restriction was applied, and the searches were not limited by language. The detailed search strategy is available in Table S6.

### 5.2. Eligibility Criteria

We considered studies to be included if they met the following criteria: (1) participants (≥18 years of age) with NDD- or DD-CKD; (2) the intervention group received dietary fiber supplementation (including prebiotics), while the control group received a placebo (e.g., maltodextrin), a low-fiber control (e.g., muffin), or continued with their habitual diet; (3) the outcomes reported on uremic toxins included at least one of the following: p-cresyl sulfate (PCS), indoxyl sulfate (IS), blood urea nitrogen (BUN), uric acid, and trimethylamine N-oxide (TMAO); (4) randomized, controlled, parallel, or crossover trial design. We excluded studies that (1) involved supplementation of synbiotic (a combination of prebiotics and probiotics) in the intervention group, (2) involved animal or in vitro experiments, and (3) were reviews, conference abstracts, meta-analyses, and responses to letters.

### 5.3. Study Outcomes

The primary outcome was the effects of dietary fiber supplementation on reducing serum uremic toxin concentrations (pre-post changes), including PCS, IS, BUN, uric acid, and TMAO, compared to the control groups. The secondary outcome was the reduction in inflammatory markers (pre-post changes), including high-sensitivity C-reactive protein (hs-CRP), interleukin-6 (IL-6), and tumor necrotic factor alpha (TNF-α), compared to the control groups.

### 5.4. Data Extraction

Two reviewers (W.W. and W.C.) independently extracted data from each study and organized it into tables. The data tables were categorized by topics, including the first author, year of publication, research country, study design, participant numbers, age and gender of participants, CKD status, dialysis vintage, intervention (i.e., fiber type, dosage, and duration), comparator, outcomes of interest (i.e., uremic toxins, inflammatory markers), and risk of bias score. Any discrepancies during data extraction were resolved through discussion with a third author (T.F.). In case of duplicated studies of the same patient population, the study reporting the higher number of participants was selected as the main data source. For studies that reported outcomes at multiple time-points, the time-point with the longest follow-up was selected. For outcomes of interest reported as continuous scales, we extracted the mean values, mean changes and their standard deviations (SDs), or the median values, median change, and their interquartile range (IQR) for each treatment group, as applicable. If the data were not provided in text or table format, we used an online graph reader tool (http://automeris.io) to extract data from graph images, ensuring accuracy through careful evaluation by the authors. For crossover studies, we planned to use data from the first period, if available. If only combined data from both periods were reported, we treated the study as a parallel study, noting the potential bias this could introduce, and interpreting the results accordingly [70].

### 5.5. Assessments of Quality and Risk of Bias

The risk of bias in each included study was also independently assessed by two reviewers (W.W. and W.C.) using the Cochrane risk-of-bias tool for randomized trials (RoB 2) [71] as recommended by the Cochrane, 2022. Any discrepancies were resolved by the third author (T.F.).

### 5.6. Data Synthesis

Data reported as median and IQR were initially converted to mean and SD using the method described by Wan et al. [72]. Additionally, for trials that reported only standard error of the mean (SEM), the SD was computed using the formula SD = SEM x √n, where “n” is the number of subjects in each group. If a study did not provide change values for uremic toxins and inflammatory markers, the mean ± SD of their levels at baseline (pre-intervention) and endpoint (post-intervention) were collected. We then calculated the pre-post changes in mean and SD for each group in the study using the following formula: mean change = mean post-intervention—mean baseline. If the SD of change was not provided, it was computed using the following formula: SD_change_ = √[(SD_baseline_^2^ + SD_final_^2^) − (2 × R × SD_baseline_ × SD_final_)] [73].

### 5.7. Statistical Analysis

All the statistical analyses were performed using Stata statistical package version 18 (StataCorp, College Station, TX, USA). Statistical significance was determined with a two-tailed *p*-value of less than 0.05. Meta-analyses were conducted including RCTs that compared ∆ in uremic toxins and inflammatory marker outcomes following dietary fiber intervention relative to control groups, using a random-effects model to estimate pooled effect sizes and 95% confidence intervals (95% CI). Since the outcome data were continuous variables reported in different units, we used SMD with 95% CI as summary statistics. Effect sizes were classified as negligible (0–0.2), small (>0.2–0.5), medium (>0.5–0.8), or large (>0.8).

Statistical heterogeneity was assessed using the I^2^ statistic and Q-test *p* value. The I^2^ index ranges between 0% and 100%, with higher values indicating greater degrees of variability across the study results. I^2^ values of 25%, 50%, and 75% have been suggested to indicate low, moderate, and high heterogeneity, respectively. To explore sources of heterogeneity, we performed pre-specified subgroup meta-analyses based on the following factors: (1) race (Asian vs. non-Asian), (2) study design (parallel vs. crossover), (3) fiber supplementation dosage (≤15 g/d vs. >15 g/d), (4) CKD status (NDD-CKD vs. DD-CKD (HD and PD)), (5) fiber type (non-starch polysaccharide (NSP), resistant oligosaccharide (RO), resistant starch (RS), and mixed), (6) intervention duration (<8 weeks vs. ≥8 weeks), (7) fiber solubility (soluble vs. non-soluble), and (8) study risk of bias (low, some concerns, and high). Meta-regressions were conducted to determine whether study characteristics accounted for the observed heterogeneity, with variables, including publication year, age of participants, total sample size, dialysis vintage, and fiber supplementation dosage included as predictors. We also performed a sensitivity analysis using the leave-one-out method, removing one study at a time to assess the robustness of meta-analysis results. To graphically represent this heterogeneity among the included studies, a forest plot was employed. Publication bias was formally evaluated using Funnel plots and the Egger test.

### 5.8. Grading the Strength of Evidence

W.W. and W.C. individually graded the strength of evidence for the reduction in uremic toxins and inflammatory markers by considering the risk of bias of each study, inconsistency of the results, indirectness of evidence, imprecision, and reporting bias following the Grading of Recommended Assessment, Development, and Evaluation approach (GRADE) [74].

## Figures and Tables

**Table 1:** Characteristics of the studies included in the systematic review.

No.	Author	Publication Year	Country	Design	No. of Patients	Mean Age (Year)	Men (%)	CKD Status	Dialysis Vintage (Year)	Intervention of Interest				Comparator	Outcomes of Interest		Risk of Bias
										Fiber Use	Type of Fiber	Daily Dose (g/day)	Duration of Intervention (Week)		Uremic Toxins	Inflammatory Markers	
1	Bliss DZ.	1996	US	Crossover	20	20–72	65	NDD	NA	Gum arabic	NSP	50	4	Pectin	BUN	NA	High
2	Ali AA.	2007	Sudan	Parallel	36	44.1	69.4	HD	19.2	Gum arabic	NSP	50	12	FF + folic	BUN, Uric	NA	High
3	Sirich TL.	2014	US	Parallel	40	56	60	HD	48	High-amylose corn starch	RS	15	6	Waxy corn starch	BUN, PCS, IS	NA	Low
4	Meksawan K.	2016	Thailand	Crossover	9	71.2	55.6	PD	17.8	FOS	RO	20	4	Sucrose	BUN	NA	High
5	Poesen R.	2016	Belgium	Crossover	40	70	70	NDD	NA	AXOS	RO	20	4	Maltodextrin	PCS, IS, TMAO	NA	Low
6	Ramos CI.	2018	Brazil	Parallel	50	57.3	54	NDD	NA	Short-chain prebiotic FOS (NutraFlora^®^)	RO	12	12	Maltodextrin	PCS, IS, BUN	hs-CRP, IL-6	High
7	Esgalhado M.	2018	Brazil	Parallel	43	54.7	58.1	HD	47.1	Hi-Maize^®^	RS	16	4	Manioc flour	PCS, IS, BUN	hs-CRP, IL-6	Some concerns
8	Khosroshari HT.	2019	Iran	Parallel	50	55.5	58	HD	59.4	HAM-RS2	RS	1st 4 week; 20, 2nd 4 week; 25	8	Waxy corn starch	PCS, IS, BUN, Uric	hs-CRP	Low
9	Laffin MR.	2019	Iran	Parallel	20	55.89	65	HD	NA	HAM-RS2	RS	1st 4 week; 20, 2nd 4 week; 25	8	Wheat flour	BUN, Uric,	IL-6	High
10	Li L.	2019	China	Crossover	21	35.48	60	PD	23.01	Inulin-type fructans	RO	10	12	Maltodextrin	PCS, IS	NA	Low
11	Esgalhado M.	2020	Brazil	Crossover	26	54.71	NA	HD	47.06	Hi-Maize^®^	RS	16	4	Manioc flour	PCS, IS	NA	Some concerns
12	de Andrade LS.	2021	Brazil	Crossover	43	52	53.5	PD	22.25	Unripe banana flour	RS	21	4	Corn starch	PCS, IS, BUN	hs-CRP, IL-6, TNF-α	High
13	Biruete A.	2021	Belgium	Crossover	12	55	50	HD	NA	Inulin (91% inulin, 9% short chain FOS)	RO	Female 10, Male 15	4	Maltodextrin	PCS, IS	NA	Low
14	He S.	2022	China	Crossover	16	37	62.5	PD	25	Mixture of inulin and oligofructose	RO	10	12	Maltodextrin	Uric	NA	Low
15	Ebrahim Z.	2022	South Africa	Parallel	59	41	42.4	NDD	NA	B-glucan prebiotics	NSP	6	14	Nil	PCS, IS	NA	Low
16	Kemp JA.	2022	Brazil	Parallel	25	53.8	56	HD	45.84	Hi-Maize^®^	RS	16	4	Manioc flour	TMAO	NA	Some concerns
17	Xiong Q.	2023	China	Crossover	22	37.3	54.5	PD	28	Inulin-type Fructans	RO	10	12	Maltodextrin	TMAO	NA	High
18	Chang L.	2023	China	Parallel	45	51.4	51.1	NDD	NA	Inulin	RO	10	12	Wheat starch	BUN, PCS, IS	hs-CRP, IL-6, TNF-α	Low
19	Fatani AMN.	2023	US	Crossover	18	39.04	55.6	HD	NA	Pea hull fiber	Mixed	9	4	Control muffin	PCS, IS, TMAO	NA	Low
20	Headley SA.	2024	US	Parallel	65	60.91	48	NDD	NA	HAM-RS2	RS	1st week; 15 then 2nd–16th; up to 33	16	Waxy corn starch	PCS, IS	IL-6, hs-CRP	Low
21	Cui Y.	2024	China	Parallel	40	68.35	55	NDD	NA	Soluble dietary fiber *	Mixed	Initial 5 g/d in daily increment to a final dose of 15 g/d	4	Maltodextrin	BUN, TMAO	IL-6, TNF-α	High

Abbreviation: AXOS, arabinoxylan oligosaccharides; BUN, blood urea nitrogen; FF, ferrous fumarate; FOS, fructo-oligosaccharide; HAM-RS2, High amylose resistant starch; Hi-Maize^®^, High-amylose maize starch; HD, hemodialysis; hs-CRP, high-sensitivity C-reactive protein; IL-6, interleukin-6; IS, indoxyl sulfate; NA, not applicable; NDD, non-dialysis dependent; NSP, non-starch polysaccharide; PD, peritoneal dialysis; PCS, p-cresyl sulfate; RO, resistant oligosaccharide; RS, resistant starch; TMAO, trimethylamine N-oxide; TNF-α, tumor necrotic factor alpha; US, United States. * Soluble fiber, including a mixture of inulin, resistant dextrin, fructo-oligosaccharide, galacto-oligosaccharides, xylo-oligosaccharides, and glucomannans.

**Table 4:** Subgroup analyses examining the effect of dietary fiber supplementation on serum IS.

Subgroup Analyses	No. of Studies	SMD (95% CI)	*p*-Values	Assessment of Heterogeneity		*p* for Interaction
				I^2^ Index	*p*-Value	
**CKD status**						0.73
NDD-CKD	3	−0.40 (−0.83 to 0.03)	0.07	43.09%	0.17	
DD-CKD	8	−0.31 (−0.59 to −0.02)	0.03	20.17%	0.27	
**Study design**						0.27
Crossover	5	−0.18 (−0.52 to 0.16)	0.30	0.30%	0.40	
Parallel	6	−0.44 (−0.73 to −0.14)	<0.01	29.97%	0.21	
**Fiber dosage**						0.72
≤15 g/d	7	−0.31 (−0.59 to −0.04)	0.03	21.85%	0.26	
>15 g/d	4	−0.41 (−0.85 to 0.04)	0.07	36.09%	0.20	
**Race**						0.66
Asian	3	−0.41 (−0.78 to −0.05)	0.03	0%	0.48	
Non-Asian	8	−0.31 (−0.61 to 0.00)	0.05	35.41%	0.15	
**Study risk of bias**						0.10
Low	7	−0.35 (−0.60 to −0.10)	0.01	5.83%	0.38	
Some concerns	2	−0.76 (−1.32 to −0.19)	0.01	15.89%	0.28	
High	2	+0.04 (−0.43 to 0.52)	0.87	0%	0.95	
**Intervention duration**						0.84
<8 week	6	−0.31 (−0.70 to 0.08)	0.12	39.17%	0.14	
≥8 week	5	−0.36 (−0.63 to −0.09)	0.01	5.55%	0.38	
**Type of fiber**						0.18
NSP	1	−0.59 (−1.11 to −0.07)	-	-		
RO	4	−0.32 (−0.65 to 0.00)	0.05	0%	0.40	
RS	5	−0.40 (−0.74 to −0.07)	0.02	14.81%	0.32	
Mixed	1	0.43 (−0.34 to 1.21)	-	-		
**Fiber solubility**						0.62
Non-water soluble	6	−0.28 (−0.66 to 0.09)	0.14	41.31%	0.13	
Water soluble	5	−0.40 (−0.68 to −0.12)	<0.01	0%	0.25	

Abbreviation: NSP, non-starch polysaccharide; RO, resistant oligosaccharide; RS, resistant starch; SMD, standardized mean difference.

## Data Availability

The original contributions presented in this study are included in the article/Supplementary Material. Further inquiries can be directed to the corresponding author(s).

## Supplementary Materials

The following supporting information can be downloaded at: https://www.mdpi.com/article/10.3390/toxins17020057/s1, Figure S1: Funnel plot of individual studies displaying the standard error by the standardized mean difference for serum PCS; Figure S2: Funnel plot of individual studies displaying the standard error by the standardized mean difference for serum IS; Figure S3: Funnel plot of individual studies displaying the standard error by the standardized mean difference for BUN; Table S1: Subgroup analyses examining the effect of dietary fiber supplementation on BUN; Table S2: Subgroup analyses examining the effect of dietary fiber supplementation on serum IL-6; Table S3: Results of meta-regression between variables and uremic toxins (i.e., serum PCS, serum IS, and BUN); Table S4: GRADE evidence profile for the effect of dietary fiber supplementation on uremic toxins and inflammatory markers among CKD patients; Table S5: PRISMA 2020 Checklist; Table S6: Keywords for article searches. Reference [75] is cited in the Supplementary Materials.
