# Supplementary material for: Effects of Dietary Fiber Supplementation on Modulating Uremic Toxins and Inflammation in Chronic Kidney Disease Patients: A Systematic Review and Meta-Analysis of Randomized Controlled Trials
Source: Toxins (Basel). 2025 Jan 26;17(2):57. doi: 10.3390/toxins17020057 (PMC11860371; doi:10.3390/toxins17020057)
Supplement: Supplementary file 1 [file toxins-17-00057-s001.zip › toxins-3432182-supplementary.pdf]

Supplementary Materials: Effects of Dietary Fiber Supplementation on Modulating Uremic Toxins and Inflammation in Chronic Kidney Disease Patients: A Systematic Review and Meta-Analysis of Randomized Controlled Trials

Wannasit Wathanavasin, Wisit Cheungpasitporn, Charat Thongprayoon and Tibor Fülöp

Table S1. Sub-group analyses examining the effect of dietary fiber supplementation on BUN.

Subgroup analyses	No. of studies	SMD (95% CI)	P-values	Assessment of heterogeneity		P for interaction
				I^2 index	P-value	
**CKD status**						0.12
NDD-CKD	4	-0.06 (-0.37 to 0.24)	0.67	0%	0.49	
DD-CKD	7	-0.41 (-0.72 to -0.09)	0.01	25.53%	0.23	
**Study design**						0.65
Crossover	3	-0.39 (-1.04 to 0.26)	0.24	61.34%	0.08	
Parallel	8	-0.23 (-0.47 to 0.01)	0.06	6.88%	0.38	
**Fiber dosage**						0.20
≤15 g/d	4	-0.10 (-0.40 to 0.21)	0.54	4.33%	0.37	
>15 g/d	7	-0.38 (-0.70 to -0.07)	0.02	25.83%	0.23	
**Race**						0.86
Asian	4	-0.23 (-0.78 to 0.31)	0.40	61.17%	0.05	
Non-Asian	7	-0.29 (-0.54 to -0.04)	0.02	0%	0.59	
**Study risk of bias**						0.79
Low	3	-0.25 (-0.60 to 0.10)	0.16	0%	0.61	
Some concerns	1	-0.05 (-0.76 to 0.65)	0.88	-	-	
High	7	-0.33 (-0.70 to 0.04)	0.08	47.59%	0.08	
**Intervention duration**						0.52
< 8 week	6	-0.19 (-0.54 to 0.16)	0.29	41.25%	0.13	
≥ 8 week	5	-0.34 (-0.64 to -0.04)	0.03	0%	0.49	
**Type of fiber**						0.25
NSP	2	-0.48 (-1.01 to 0.06)	0.08	8.47%	0.30	
RO	3	-0.40 (-1.02 to 0.21)	0.20	56.99%	0.10	
RS	5	-0.25 (-0.53 to 0.04)	0.10	0%	0.56	
Mixed	1	0.32 (-0.30 to 0.95)	0.31	-	-	
**Fiber solubility**						0.15
Non-water soluble	5	-0.25 (-0.53 to 0.04)	0.10	0%	0.56	
Water soluble	6	-0.41 (-0.79 to -0.03)	0.04	33.39%	0.20	

Abbreviation: NSP, non-starch polysaccharide; RO, resistant oligosaccharide; RS, resistant starch; SMD standardized mean difference.

Table S2. Sub-group analyses examining the effect of dietary fiber supplementation on serum IL-6.

Subgroup analyses	No. of studies	SMD (95% CI)	P-values	Assessment of heterogeneity		P for interaction
				I^2 index	P-value	
**CKD status**						0.84
NDD-CKD	3	-0.50 (-0.85 to -0.15)	<0.01	0%	0.67	
DD-CKD	4	-0.44 (-0.97 to 0.10)	0.11	55.84%	0.08	
**Study design**						0.96
Crossover	2	-0.45 (-1.57 to 0.66)	0.43	82.78%	0.02	
Parallel	5	-0.48 (-0.78 to -0.19)	<0.01	0%	0.79	
**Fiber dosage**						0.84
≤15 g/d	3	-0.50 (-0.85 to -0.15)	<0.01	0%	0.67	
>15 g/d	4	-0.44 (-0.97 to 0.10)	0.11	55.84%	0.08	
**Race**						0.78
Asian	2	-0.51 (-0.95 to -0.08)	0.02	0%	0.37	
Non-Asian	5	-0.43 (-0.83 to -0.03)	0.04	42.21%	0.14	
**Study risk of bias**						0.42
Low	1	-0.70 (-1.31 to -0.10)	0.02	-	-	
Some concerns	2	-0.63 (-1.44 to 0.18)	0.13	59.48%	0.12	
High	4	-0.29 (-0.62 to 0.05)	0.09	9.72%	0.34	
**Intervention duration**						0.34
< 8 week	4	-0.33 (-0.79 to 0.12)	0.15	48.68%	0.12	
≥ 8 week	3	-0.62 (-1.00 to -0.24)	<0.01	0%	0.81	
**Type of fiber**						0.75
NSP	2	-0.59 (-1.01 to -0.17)	0.01	0%	0.60	
RO	4	-0.44 (-0.97 to 0.10)	0.11	55.84%	0.08	
RS	1	-0.31 (-0.93 to 0.31)	0.33	-	-	
**Fiber solubility**						0.84
Non-water soluble	4	-0.44 (-0.97 to 0.10)	0.11	55.84%	0.08	
Water soluble	3	-0.50 (-0.85 to -0.15)	<0.01	0%	0.67	

Abbreviation: NSP, non-starch polysaccharide; RO, resistant oligosaccharide; RS, resistant starch; SMD standardized mean difference.

Table S3. Results of meta-regression between variables and uremic toxins (i.e., serum PCS, serum IS, and BUN).

Variable	Beta	SE	95% CI	z	P-value	I^2 (%)	R^2 (%)
**Serum PCS**							
Age of participants	-0.006	0.014	-0.03 to 0.02	-0.46	0.647	0	0
Study sample size	-0.015	0.007	-0.03 to -0.001	-2.14	0.029	0	0
Year of publication	+0.004	0.039	-0.71 to 0.08	0.11	0.910	0	0
Fiber dosage	+0.005	0.018	-0.03 to 0.04	0.28	0.778	0	0
Duration of fiber supplementation	-0.044	0.025	-0.09 to 0.01	-1.73	0.083	0	0
Dialysis vintage	-0.007	0.011	-0.03 to 0.01	-0.64	0.520	0	0
**Serum IS**							
Age of participants	- 0.001	0.016	-0.03 to 0.03	-0.01	0.949	27.87	0
Study sample size	- 0.006	0.008	-0.02 to 0.01	-0.70	0.483	24.18	0
Year of publication	+0.133	0.047	-0.08 to 0.01	0.28	0.776	27.81	0
Fiber dosage	+0.011	0.022	-0.03 to 0.05	0.51	0.609	25.10	0
Duration of fiber supplementation	- 0.016	0.030	-0.08 to 0.04	-0.53	0.594	25.57	0
Dialysis vintage	-0.002	0.012	-0.02 to 0.02	-0.15	0.880	15.37	0
**BUN**							
Age of participants	+0.002	0.020	-0.04 to 0.04	0.12	0.906	36.60	0
Study sample size	+0.017	0.009	-0.001 to 0.04	1.85	0.064	2.76	89.70
Year of publication	+0.018	0.014	-0.01 to 0.05	1.29	0.198	16.15	28.29
Fiber dosage	-0.011	0.009	-0.03 to 0.01	-1.22	0.223	18.17	18.25
Duration of fiber supplementation	-0.022	0.036	-0.09 to 0.05	-0.62	0.534	26.08	0
Dialysis vintage	+0.008	0.012	-0.02 to 0.03	0.70	0.486	43.22	0

Abbreviation: BUN, blood urea nitrogen; IS, indoxyl sulfate; PCS, p-cresyl sulfate

Table S4. GRADE evidence profile for the effect of dietary fiber supplementation on uremic toxins and inflammatory markers among CKD patients.

Certainty assessment							Certainty	Importance
**№ of studies**	**Study design**	**Risk of bias**	**Inconsistency**	**Indirectness**	**Imprecision**	**Other considerations**		
**Serum PCS**								
11	randomised trials	not serious	not serious	not serious	not serious	none	⊕⊕⊕⊕ High	IMPORTANT
**Serum IS**								
11	randomised trials	not serious	not serious	not serious	not serious	none	⊕⊕⊕⊕ High	IMPORTANT
**BUN**								
11	randomised trials	serious	not serious	not serious	not serious	publication bias strongly suspected	⊕⊕◯◯ Low	IMPORTANT
**Serum TMAO**								
4	randomised trials	serious	not serious	not serious	serious	publication bias strongly suspected	⊕◯◯◯ Very low	IMPORTANT
**Serum uric acid**								
4	randomised trials	serious	not serious	not serious	serious	publication bias strongly suspected	⊕◯◯◯ Very low	IMPORTANT
**Serum IL-6**								
7	randomised trials	serious	not serious	not serious	not serious	none	⊕⊕⊕◯ Moderate	IMPORTANT
**Serum hs-CRP**								
5	randomised trials	serious	not serious	not serious	serious	publication bias strongly suspected	⊕◯◯◯ Very low	IMPORTANT
**Serum TNF-alpha**								

Certainty assessment							Certainty	Importance
**№ of studies**	**Study design**	**Risk of bias**	**Inconsistency**	**Indirectness**	**Imprecision**	**Other considerations**		
4	randomised trials	serious	serious	not serious	serious	publication bias strongly suspected	⨁◯◯◯ Very low	IMPORTANT

Abbreviation: BUN, blood urea nitrogen; CI, confident interval; hs-CRP, high sensitivity C-reactive protein; IL-6, interleukin-6; IS, indoxyl sulfate; PCS, p-cresyl sulfate; SCFAs; TMAO, trimethylamine N-oxide; TNF-α, tumor necrotic factor alpha.

Table S5. PRISMA 2020 Checklist.

Section and Topic	Item #	Checklist item	Location where item is reported
**TITLE**			
Title	1	Identify the report as a systematic review.	1
**ABSTRACT**			
Abstract	2	See the PRISMA 2020 for Abstracts checklist.	1
**INTRODUCTION**			
Rationale	3	Describe the rationale for the review in the context of existing knowledge.	2-3
Objectives	4	Provide an explicit statement of the objective(s) or question(s) the review addresses.	3
**METHODS**			
Eligibility criteria	5	Specify the inclusion and exclusion criteria for the review and how studies were grouped for the syntheses.	18
Information sources	6	Specify all databases, registers, websites, organisations, reference lists and other sources searched or consulted to identify studies. Specify the date when each source was last searched or consulted.	17 and references
Search strategy	7	Present the full search strategies for all databases, registers and websites, including any filters and limits used.	17, Table S6
Selection process	8	Specify the methods used to decide whether a study met the inclusion criteria of the review, including how many reviewers screened each record and each report retrieved, whether they worked independently, and if applicable, details of automation tools used in the process.	18
Data collection process	9	Specify the methods used to collect data from reports, including how many reviewers collected data from each report, whether they worked independently, any processes for obtaining or confirming data from study investigators, and if applicable, details of automation tools used in the process.	18
Data items	10a	List and define all outcomes for which data were sought. Specify whether all results that were compatible with each outcome domain in each study were sought (e.g. for all measures, time points, analyses), and if not, the methods used to decide which results to collect.	18
	10b	List and define all other variables for which data were sought (e.g. participant and intervention characteristics, funding sources). Describe any assumptions made about any missing or unclear information.	18
Study risk of bias assessment	11	Specify the methods used to assess risk of bias in the included studies, including details of the tool(s) used, how many reviewers assessed each study and whether they worked independently, and if applicable, details of automation tools used in the process.	18
Effect measures	12	Specify for each outcome the effect measure(s) (e.g. risk ratio, mean difference) used in the synthesis or presentation of results.	19

Section and Topic	Item #	Checklist item	Location where item is reported
Synthesis methods	13a	Describe the processes used to decide which studies were eligible for each synthesis (e.g. tabulating the study intervention characteristics and comparing against the planned groups for each synthesis (item #5)).	18
	13b	Describe any methods required to prepare the data for presentation or synthesis, such as handling of missing summary statistics, or data conversions.	18-19
	13c	Describe any methods used to tabulate or visually display results of individual studies and syntheses.	19
	13d	Describe any methods used to synthesize results and provide a rationale for the choice(s). If meta-analysis was performed, describe the model(s), method(s) to identify the presence and extent of statistical heterogeneity, and software package(s) used.	19
	13e	Describe any methods used to explore possible causes of heterogeneity among study results (e.g. subgroup analysis, meta-regression).	19
	13f	Describe any sensitivity analyses conducted to assess robustness of the synthesized results.	19
Reporting bias assessment	14	Describe any methods used to assess risk of bias due to missing results in a synthesis (arising from reporting biases).	18
Certainty assessment	15	Describe any methods used to assess certainty (or confidence) in the body of evidence for an outcome.	19
**RESULTS**			
Study selection	16a	Describe the results of the search and selection process, from the number of records identified in the search to the number of studies included in the review, ideally using a flow diagram.	3, Figure 2
	16b	Cite studies that might appear to meet the inclusion criteria, but which were excluded, and explain why they were excluded.	3-4, Figure 2
Study characteristics	17	Cite each included study and present its characteristics.	3-4, Figure 2, Table 1
Risk of bias in studies	18	Present assessments of risk of bias for each included study.	7, Figure 3A, 3B
Results of individual studies	19	For all outcomes, present, for each study: (a) summary statistics for each group (where appropriate) and (b) an effect estimate and its precision (e.g. confidence/credible interval), ideally using structured tables or plots.	Figure 4-5 Table 2-4, S1-2
Results of syntheses	20a	For each synthesis, briefly summarise the characteristics and risk of bias among contributing studies.	3-4, 7, Table 1

Section and Topic	Item #	Checklist item	Location where item is reported
	20b	Present results of all statistical syntheses conducted. If meta-analysis was done, present for each the summary estimate and its precision (e.g. confidence/credible interval) and measures of statistical heterogeneity. If comparing groups, describe the direction of the effect.	7-12, Figure 4-5, Table 2
	20c	Present results of all investigations of possible causes of heterogeneity among study results.	7-12, Table 3-4, Table S1-2
	20d	Present results of all sensitivity analyses conducted to assess the robustness of the synthesized results.	12, Table S3
Reporting biases	21	Present assessments of risk of bias due to missing results (arising from reporting biases) for each synthesis assessed.	12-13, Figure S1-3
Certainty of evidence	22	Present assessments of certainty (or confidence) in the body of evidence for each outcome assessed.	12-13, Table S4
**DISCUSSION**			
Discussion	23a	Provide a general interpretation of the results in the context of other evidence.	13
	23b	Discuss any limitations of the evidence included in the review.	13-17
	23c	Discuss any limitations of the review processes used.	17
	23d	Discuss implications of the results for practice, policy, and future research.	17
**OTHER INFORMATION**			
Registration and protocol	24a	Provide registration information for the review, including register name and registration number, or state that the review was not registered.	17
	24b	Indicate where the review protocol can be accessed, or state that a protocol was not prepared.	17
	24c	Describe and explain any amendments to information provided at registration or in the protocol.	N/A
Support	25	Describe sources of financial or non-financial support for the review, and the role of the funders or sponsors in the review.	20
Competing interests	26	Declare any competing interests of review authors.	20
Availability of data, code and other materials	27	Report which of the following are publicly available and where they can be found: template data collection forms; data extracted from included studies; data used for all analyses; analytic code; any other materials used in the review.	20

From: Page MJ, McKenzie JE, Bossuyt PM, Boutron I, Hoffmann TC, Mulrow CD, et al. The PRISMA 2020 statement: an updated guideline for reporting systematic reviews. BMJ 2021;372:n71. doi:

10.1136/bmj.n71

For more information, visit: http://www.prisma-statement.org/

Table S6. Keywords for article searches.

Keywords	No. of articles
Pubmed	
Population of interest<br>"renal insufficiency, chronic"[MeSH Terms] OR ("renal"[All Fields] AND "insufficiency"[All Fields] AND "chronic"[All Fields]) OR "chronic renal insufficiency"[All Fields] OR ("chronic"[All Fields] AND "kidney"[All Fields] AND "disease"[All Fields]) OR "chronic kidney disease"[All Fields] OR "CKD"[All Fields] OR ("kidney failure, chronic"[MeSH Terms] OR ("kidney"[All Fields] AND "failure"[All Fields] AND "chronic"[All Fields]) OR "chronic kidney failure"[All Fields] OR ("chronic"[All Fields] AND "renal"[All Fields] AND "failure"[All Fields]) OR "chronic renal failure"[All Fields]) OR "CRF"[All Fields] OR ("renal insufficiency"[MeSH Terms] OR ("renal"[All Fields] AND "insufficiency"[All Fields]) OR "renal insufficiency"[All Fields]) OR ("kidney failure, chronic"[MeSH Terms] OR ("kidney"[All Fields] AND "failure"[All Fields] AND "chronic"[All Fields]) OR "chronic kidney failure"[All Fields] OR "esrd"[All Fields]) OR "eskd"[All Fields] OR ("renal replacement therapy"[MeSH Terms] OR ("renal"[All Fields] AND "replacement"[All Fields] AND "therapy"[All Fields]) OR "renal replacement therapy"[All Fields]) OR ("dialysance"[All Fields] OR "dialysances"[All Fields] OR "dialysation"[All Fields] OR "dialysator"[All Fields] OR "dialysators"[All Fields] OR "dialyse"[All Fields] OR "dialysed"[All Fields] OR "dialyser"[All Fields] OR "dialysers"[All Fields] OR "dialysing"[All Fields] OR "dialysis solutions"[Pharmacological Action] OR "dialysis solutions"[MeSH Terms] OR ("dialysis"[All Fields] AND "solutions"[All Fields]) OR "dialysis solutions"[All Fields] OR "dialysate"[All Fields] OR "dialysates"[All Fields] OR "dialyzate"[All Fields] OR "dialyzates"[All Fields] OR "dialysis"[MeSH Terms] OR "dialysis"[All Fields] OR "dialyses"[All Fields] OR "dialyzability"[All Fields] OR "dialyzable"[All Fields] OR "dialyzation"[All Fields] OR "dialyze"[All Fields] OR "dialyzed"[All Fields] OR "dialyzer"[All Fields] OR "dialyzer s"[All Fields] OR "dialyzers"[All Fields] OR "dialyzing"[All Fields] OR "renal dialysis"[MeSH Terms] OR ("renal"[All Fields] AND "dialysis"[All Fields]) OR "renal dialysis"[All Fields]) OR ("haemodialysis"[All Fields] OR "renal dialysis"[MeSH Terms] OR ("renal"[All Fields] AND "dialysis"[All Fields]) OR "renal dialysis"[All Fields] OR "hemodialysis"[All Fields]) OR ("haemodialysis"[All Fields] OR "renal dialysis"[MeSH Terms] OR ("renal"[All Fields] AND "dialysis"[All Fields]) OR "renal dialysis"[All Fields] OR "hemodialysis"[All Fields]) OR ("peritoneal dialysis"[MeSH Terms] OR ("peritoneal"[All Fields] AND "dialysis"[All Fields]) OR "peritoneal dialysis"[All Fields])<br>**AND**<br>_Intervention of interest_<br>"dietary fibre"[All Fields] OR "dietary fiber"[MeSH Terms] OR ("dietary"[All Fields] AND "fiber"[All Fields]) OR "dietary fiber"[All Fields] OR ("dietary fibres"[All Fields] OR "dietary fiber"[MeSH Terms]	**508**

OR ("dietary"[All Fields] AND "fiber"[All Fields]) OR "dietary fiber"[All Fields] OR ("dietary"[All Fields] AND "fibers"[All Fields]) OR "dietary fibers"[All Fields]) OR "fiber*"[All Fields] OR ("dietary fiber"[MeSH Terms] OR ("dietary"[All Fields] AND "fiber"[All Fields]) OR "dietary fiber"[All Fields] OR ("wheat"[All Fields] AND "bran"[All Fields]) OR "wheat bran"[All Fields]) OR ("dietary fiber"[MeSH Terms] OR ("dietary"[All Fields] AND "fiber"[All Fields]) OR "dietary fiber"[All Fields] OR ("bran"[All Fields] AND "wheat"[All Fields]) OR "bran wheat"[All Fields]) OR ("dietary fiber"[MeSH Terms] OR ("dietary"[All Fields] AND "fiber"[All Fields]) OR "dietary fiber"[All Fields] OR ("brans"[All Fields] AND "wheat"[All Fields]) OR "brans wheat"[All Fields]) OR ("dietary fiber"[MeSH Terms] OR ("dietary"[All Fields] AND "fiber"[All Fields]) OR "dietary fiber"[All Fields] OR ("wheat"[All Fields] AND "brans"[All Fields]) OR "wheat brans"[All Fields]) OR "roughage*"[All Fields] OR ("lignin"[MeSH Terms] OR "lignin"[All Fields] OR "lignins"[All Fields] OR "lignin s"[All Fields]) OR ("cellulose"[MeSH Terms] OR "cellulose"[All Fields] OR "celluloses"[All Fields] OR "cellulosic"[All Fields] OR "cellulosics"[All Fields]) OR ("arabinoxylan"[Supplementary Concept] OR "arabinoxylan"[All Fields] OR "arabinoxylans"[All Fields]) OR ("inulin"[MeSH Terms] OR "inulin"[All Fields] OR "inuline"[All Fields] OR "inulins"[All Fields]) OR "b-Glucan"[All Fields] OR ("guar gum"[Supplementary Concept] OR "guar gum"[All Fields]) OR ("gum arabic"[MeSH Terms] OR ("gum"[All Fields] AND "arabic"[All Fields]) OR "gum arabic"[All Fields] OR ("gum"[All Fields] AND "acacia"[All Fields]) OR "gum acacia"[All Fields]) OR ("pectin s"[All Fields] OR "pectinate"[All Fields] OR "pectinates"[All Fields] OR "pectinic"[All Fields] OR "pectins"[MeSH Terms] OR "pectins"[All Fields] OR "pectin"[All Fields]) OR ("psyllium"[MeSH Terms] OR "psyllium"[All Fields]) OR "Fructo-oligosaccharides"[All Fields] OR ("resistant starch"[MeSH Terms] OR ("resistant"[All Fields] AND "starch"[All Fields]) OR "resistant starch"[All Fields])<br>**AND**<br><u>*Outcomes of interest*</u><br>(("uraemic"[All Fields] OR "uraemics"[All Fields] OR "uremic"[All Fields] OR "uremics"[All Fields]) AND "toxin*"[All Fields]) OR ("indoxyl sulphate"[All Fields] OR "indican"[MeSH Terms] OR "indican"[All Fields] OR ("indoxyl"[All Fields] AND "sulfate"[All Fields]) OR "indoxyl sulfate"[All Fields]) OR ("indoxyl sulphate"[All Fields] OR "indican"[MeSH Terms] OR "indican"[All Fields] OR ("indoxyl"[All Fields] AND "sulfate"[All Fields]) OR "indoxyl sulfate"[All Fields]) OR ("indoxyl"[Supplementary Concept] OR "indoxyl"[All Fields] OR "indoxyls"[All Fields]) OR ("p-cresyl"[All Fields] AND ("bisulfate"[All Fields] OR "bisulphate"[All Fields] OR "dihydrochloride"[All Fields] OR "dihydrochlorides"[All Fields] OR "sulfatated"[All Fields] OR "sulfatation"[All Fields] OR "sulfate s"[All Fields] OR "sulfated"[All Fields] OR "sulfates"[MeSH Terms] OR "sulfates"[All Fields] OR "sulfate"[All Fields] OR "sulphate"[All Fields] OR "sulfating"[All Fields] OR "sulfation"[All Fields] OR "sulfations"[All Fields] OR "sulphated"[All Fields] OR "sulphates"[All Fields] OR "sulphation"[All Fields])) OR "p-cresyl"[All Fields] OR ("4 cresol"[Supplementary Concept] OR "4 cresol"[All Fields] OR	

"p cresol"[All Fields]) OR ("p-cresyl"[All Fields] AND ("bisulfate"[All Fields] OR "bisulphate"[All Fields] OR "dihydrochloride"[All Fields] OR "dihydrochlorides"[All Fields] OR "sulfatated"[All Fields] OR "sulfatation"[All Fields] OR "sulfate s"[All Fields] OR "sulfated"[All Fields] OR "sulfates"[MeSH Terms] OR "sulfates"[All Fields] OR "sulfate"[All Fields] OR "sulphate"[All Fields] OR "sulfating"[All Fields] OR "sulfation"[All Fields] OR "sulfations"[All Fields] OR "sulphated"[All Fields] OR "sulphates"[All Fields] OR "sulphation"[All Fields])) OR ("blood urea nitrogen"[MeSH Terms] OR ("blood"[All Fields] AND "urea"[All Fields] AND "nitrogen"[All Fields]) OR "blood urea nitrogen"[All Fields]) OR "uric"[All Fields] OR ("trimethyloxamine"[Supplementary Concept] OR "trimethyloxamine"[All Fields] OR "trimethylamine oxide"[All Fields]) OR ("trimethyloxamine"[Supplementary Concept] OR "trimethyloxamine"[All Fields] OR "trimethylamine n oxide"[All Fields]) OR ("trimethyloxamine"[Supplementary Concept] OR "trimethyloxamine"[All Fields] OR "trimethylammonium oxide"[All Fields]) OR ("trimethyloxamine"[Supplementary Concept] OR "trimethyloxamine"[All Fields] OR "tmao"[All Fields])	
Scopus	
Population of interest<br>(TITLE-ABS-KEY (chronic AND kidney AND disease) OR TITLE-ABS-KEY (ckd) OR TITLE-ABS-KEY (chronic AND renal AND failure) OR TITLE-ABS-KEY (crf) OR TITLE-ABS-KEY (renal AND insufficiency) OR TITLE-ABS-KEY (esrd) OR TITLE-ABS-KEY (eskd) OR TITLE-ABS-KEY (renal AND replacement AND therapy) OR TITLE-ABS-KEY (dialysis) OR TITLE-ABS-KEY (hemodialysis) OR TITLE-ABS-KEY (haemodialysis) OR TITLE-ABS-KEY (peritoneal AND dialysis))<br>**AND**<br>*Intervention of interest*<br>(TITLE-ABS-KEY (dietary AND fiber) OR TITLE-ABS KEY (dietary AND fibers) OR TITLE-ABS-KEY (fiber*) OR TITLE-ABS-KEY (wheat AND bran) OR TITLE-ABS KEY (bran, AND wheat) OR TITLE-ABS-KEY (wheat AND brans) OR TITLE-ABS-KEY (roughage*) OR TITLE-ABS-KEY (lignin) OR TITLE-ABS-KEY (cellulose) OR TITLE-ABS-KEY (arabinoxylan) OR TITLE-ABS-KEY (inulin) OR TITLE-ABS-KEY (b-glucan) OR TITLE-ABS-KEY (guar AND gum) OR TITLE-ABS-KEY (gum AND acacia) OR TITLE-ABS-KEY (pectin) OR TITLE-ABS-KEY (psyllium) OR TITLE-ABS-KEY (fructo-oligosaccharides) OR TITLE-ABS-KEY (resistant AND starch))<br>**AND**<br>*Outcomes of interest*<br>((TITLE-ABS-KEY (uremic AND toxin*) OR TITLE-ABS-KEY (indoxyl AND sulfate) OR TITLE-ABS-KEY (indoxyl AND sulphate) OR TITLE-ABS-KEY (indoxyl) OR TITLE-ABS-KEY (p-cresyl AND sulphate) OR TITLE-ABS-KEY (p-cresyl) OR TITLE-ABS-KEY (p-cresol) OR TITLE-	**827**

ABS-KEY (p AND cresyl AND sulfate) OR TITLE-ABS-KEY (blood AND urea AND nitrogen) OR TITLE-ABS-KEY (uric) OR TITLE-ABS-KEY (trimethylamine AND oxide) OR TITLE-ABS-KEY (trimethylamine AND n-oxide) OR TITLE-ABS-KEY (trimethylammonium AND oxide) OR TITLE-ABS-KEY (tmao))	
CENTRAL	**142**
Population of interest<br>chronic kidney disease OR CKD OR chronic renal failure OR CRF OR renal insufficiency OR esrd OR eskd OR renal replacement therapy OR dialysis OR hemodialysis OR haemodialysis OR peritoneal dialysis<br>AND<br>*Intervention of interest*<br>Dietary Fiber OR Dietary Fibers OR Fiber* OR Wheat Bran OR Bran, Wheat OR Brans, Wheat OR Wheat Brans OR Roughage* OR Lignin OR Cellulose OR Arabinoxylan OR Inulin OR b-Glucan OR Guar gum OR Gum acacia OR Pectin OR Psyllium OR Fructo-oligosaccharides OR Resistant starch<br>*Outcomes of interest*<br>uremic toxin* OR indoxyl sulfate OR indoxyl sulphate OR indoxyl OR p-cresyl sulphate OR p-cresyl OR p-cresol OR p-cresyl sulfate OR blood urea nitrogen OR uric OR trimethylamine oxide OR trimethylamine N-oxide OR trimethylammonium oxide OR TMAO	

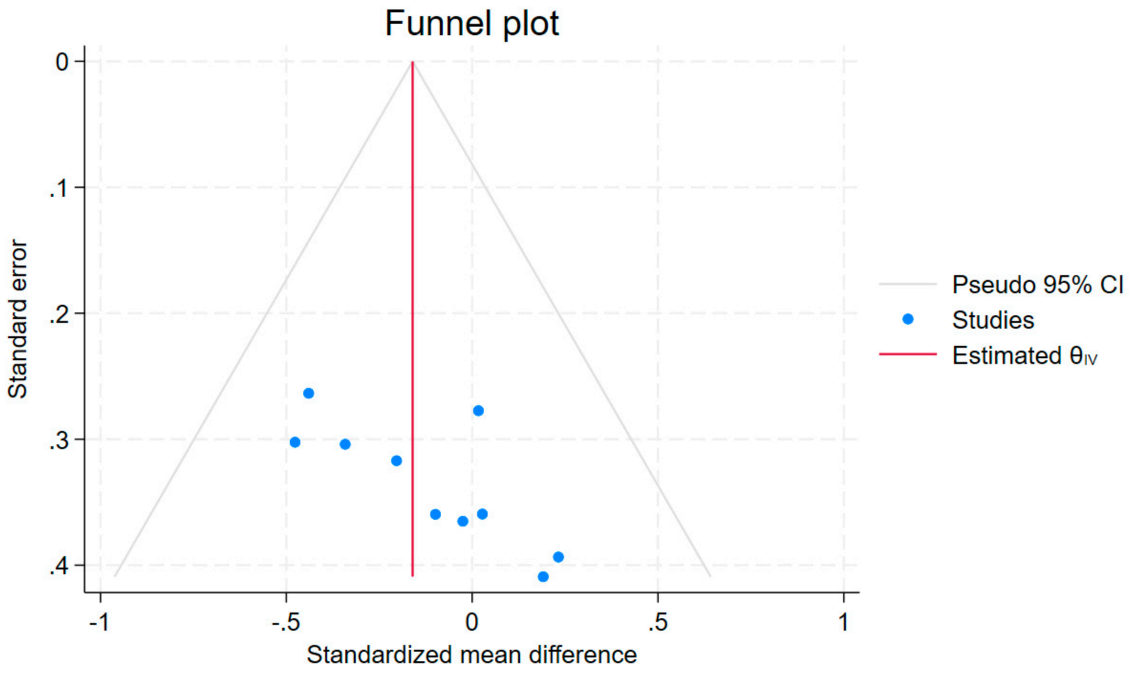

Figure S1. Funnel plot of individual studies displaying the standard error by the standardized mean difference for serum PCS.

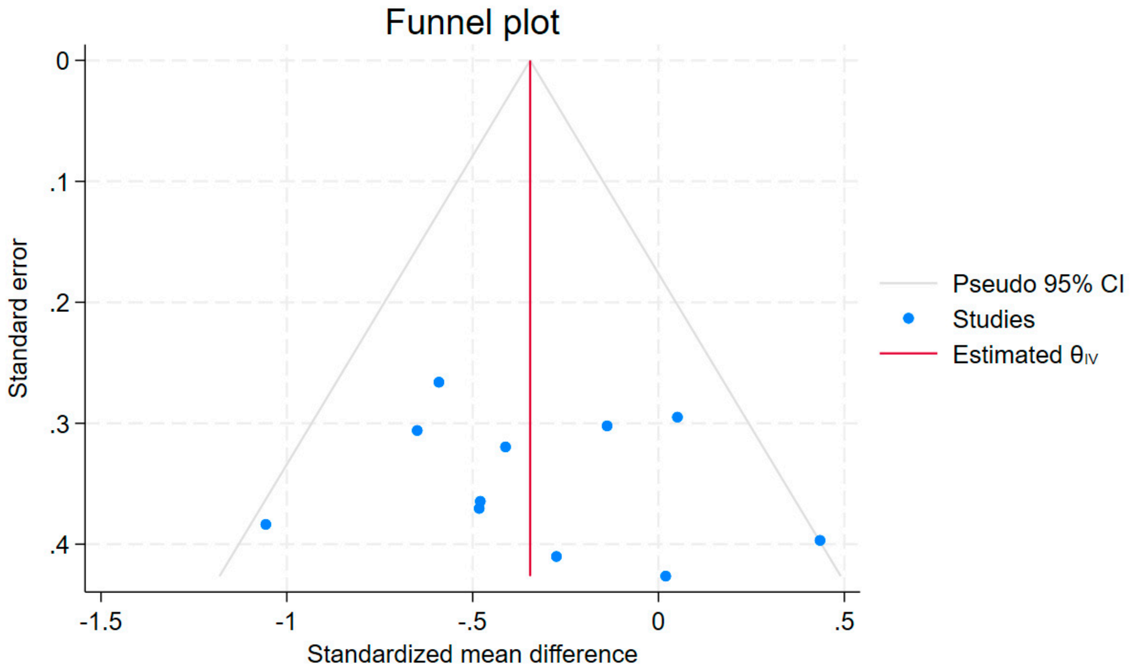

Figure S2. Funnel plot of individual studies displaying the standard error by the standardized mean difference for serum IS.

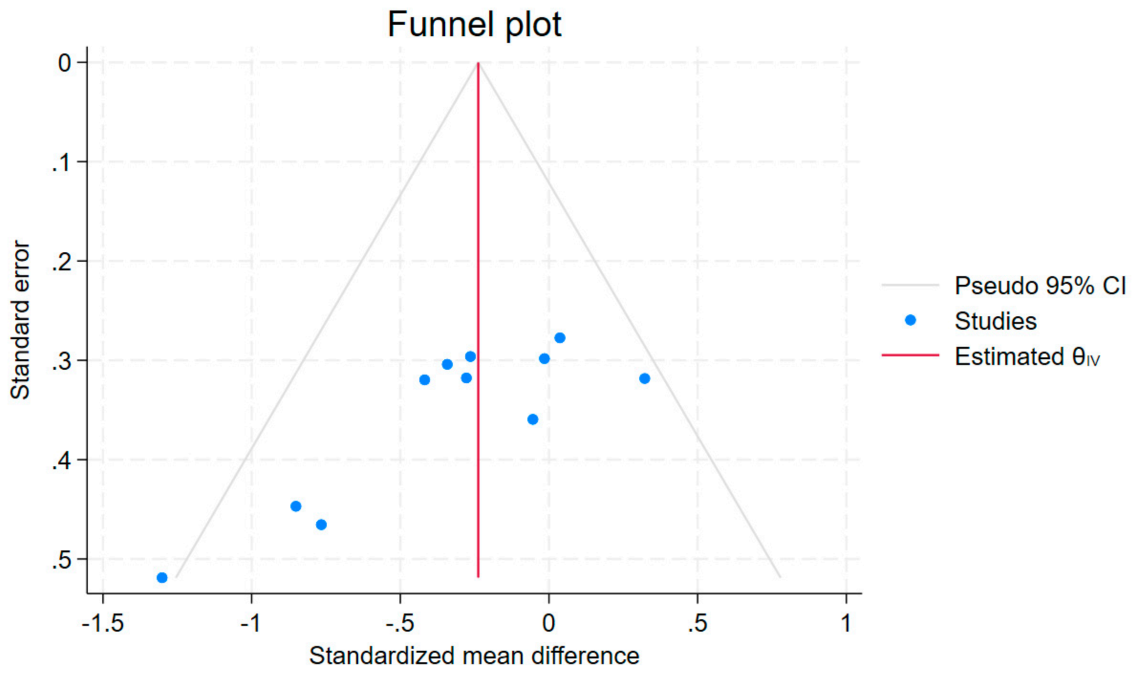

Figure S3. Funnel plot of individual studies displaying the standard error by the standardized mean difference for BUN.
